# Tracking Electron Uptake from a Cathode into *Shewanella* Cells: Implications for Energy Acquisition from Solid-Substrate Electron Donors

**DOI:** 10.1128/mBio.02203-17

**Published:** 2018-02-27

**Authors:** Annette R. Rowe, Pournami Rajeev, Abhiney Jain, Sahand Pirbadian, Akihiro Okamoto, Jeffrey A. Gralnick, Mohamed Y. El-Naggar, Kenneth H. Nealson

**Affiliations:** a Department of Earth Sciences, University of Southern California, Los Angeles, California, USA; b Department of Microbiology, University of Minnesota, St. Paul, Minnesota, USA; c BioTechnology Institute, University of Minnesota, St. Paul, Minnesota, USA; d Department of Physics and Astronomy, University of Southern California, Los Angeles, California, USA; e Global Research Center for Environment and Energy Based on Nanomaterials Science, National Institute for Materials Science (NIMS), Tsukuba, Ibaraki, Japan; f Department of Biological Sciences, University of Southern California, Los Angeles, California, USA; g Department of Chemistry, University of Southern California, Los Angeles, California, USA; University of California, Irvine

**Keywords:** electron uptake, energy acquisition, reverse electron transport, *Shewanella*, systems biology

## Abstract

While typically investigated as a microorganism capable of extracellular electron transfer to minerals or anodes, *Shewanella oneidensis* MR-1 can also facilitate electron flow from a cathode to terminal electron acceptors, such as fumarate or oxygen, thereby providing a model system for a process that has significant environmental and technological implications. This work demonstrates that cathodic electrons enter the electron transport chain of *S. oneidensis* when oxygen is used as the terminal electron acceptor. The effect of electron transport chain inhibitors suggested that a proton gradient is generated during cathode oxidation, consistent with the higher cellular ATP levels measured in cathode-respiring cells than in controls. Cathode oxidation also correlated with an increase in the cellular redox (NADH/FMNH_2_) pool determined with a bioluminescence assay, a proton uncoupler, and a mutant of proton-pumping NADH oxidase complex I. This work suggested that the generation of NADH/FMNH_2_ under cathodic conditions was linked to reverse electron flow mediated by complex I. A decrease in cathodic electron uptake was observed in various mutant strains, including those lacking the extracellular electron transfer components necessary for anodic-current generation. While no cell growth was observed under these conditions, here we show that cathode oxidation is linked to cellular energy acquisition, resulting in a quantifiable reduction in the cellular decay rate. This work highlights a potential mechanism for cell survival and/or persistence on cathodes, which might extend to environments where growth and division are severely limited.

## IMPORTANCE

The majority of our knowledge of the physiology of extracellular electron transfer derives from studies of electrons moving to the exterior of the cell. The physiological mechanisms and/or consequences of the reverse processes are largely uncharacterized. This report demonstrates that when coupled to oxygen reduction, electrode oxidation can result in cellular energy acquisition. This respiratory process has potentially important implications for how microorganisms persist in energy-limited environments, such as reduced sediments under changing redox conditions. From an applied perspective, this work has important implications for microbially catalyzed processes on electrodes, particularly with regard to understanding models of cellular conversion of electrons from cathodes to microbially synthesized products.

## INTRODUCTION

Electromicrobiology involves extracellular electron transfer (EET) between solid-phase electron-active redox compounds and microorganisms. Study of these interactions using electrochemical techniques has provided fundamental insights into microbial physiology and spawned a variety of microbe-electrode-driven applied technologies (1–4). These applications include energy recovery from waste treatment, bioremediation, and, more recently, electrosynthesis—the conversion of electrical energy to microbially synthesized products (4). The mechanisms (and variations) of outward EET (utilized for the reduction of solid substrates) are well understood for two model systems, *Shewanella* and *Geobacter*. In contrast, little is known about the mechanisms of inward EET (utilized during the oxidation of solid substrates), even in these model systems. Because of this, the technological, ecological, and environmental implications of these microbially mediated processes remain poorly understood.

**FIG 1:**
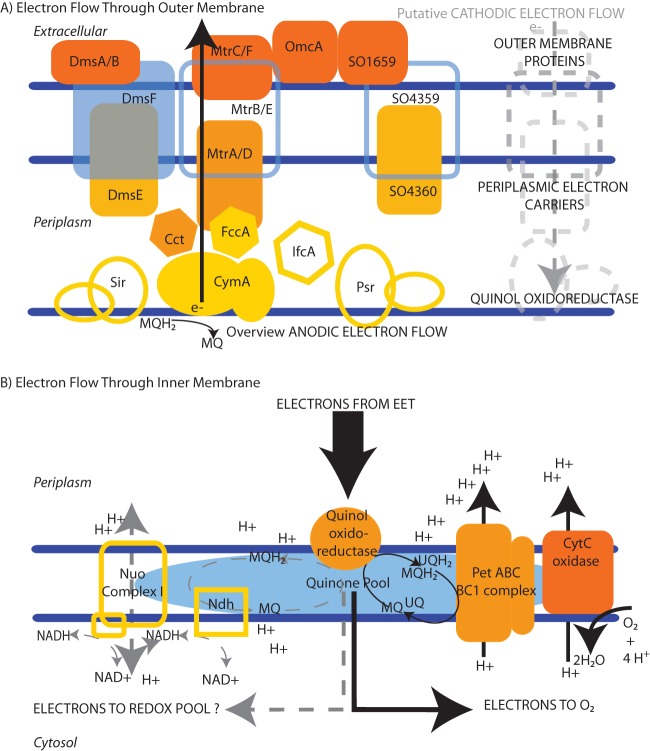
Schematic representation of MR-1 membrane proteins potentially involved in cathodic electron flow. Traditional anodic electron flow is indicated via black arrows. The potential cytochromes and/or flavochromes involved in cathodic electron flow are illustrated. (A) The outer membranes of periplasmic components tested via mutant studies are diagramed as filled shapes (details are listed in Table 1), and other known, though not tested, cytochrome-containing proteins are outlined. (B) The potential for reverse electron flow to NADH using proton motive force is also illustrated (via the reversibility of complex I-Nuo). Electron flow once electrons reach the inner membrane is diagramed as passing from the quinone pool to a terminal cytochrome oxidase and eventually oxygen.

The mechanisms of EET from the interior of a cell to external electron acceptors have been extensively characterized in the gammaproteobacterial *Shewanella oneidensis* strain MR-1. Under anaerobic conditions with an organic acid electron donor and in the presence of a suitable sink for electrons on the cell exterior, electrons from the *S. oneidensis* MR-1 inner membrane quinone pool are transferred to the inner membrane-linked tetraheme cytochrome CymA (5, 6). Electron transfer to the cell exterior is thought to depend on protein-protein interactions between CymA and periplasmic electron-carrying proteins, such as the small tetraheme cytochrome (Cct) or the flavocytochrome fumarate reductase FccA (7–9). Cct and FccA likely interact with the Mtr EET respiratory pathway through MtrA, a periplasmic decaheme cytochrome (9). MtrA helps to traffic electrons across the outer membrane via interactions with the MtrB porin and with decaheme lipoprotein cytochromes (MtrC, OmcA) localized to the exterior of the outer membrane (10). These complexes (illustrated in Fig. 1A) have been shown to be involved in electron transfer (either directly or indirectly) to solid substrates, such as solid-state electrodes, and manganese or iron (oxy)hydroxides (11).

It has been demonstrated that mineral-reducing microbes like *Shewanella*, as well as anode reduction, can facilitate cathodic reactions, i.e., the transference of electrons from an electrode to a more oxidized terminal electron acceptor (4, 12–15). Under anaerobic conditions in *S. oneidensis* MR-1, this process can be coupled to fumarate reduction and has been proposed to result from a reversal of the electron transport pathways that function in anode reductions (16, 17). Notably, this is consistent with the reversibility demonstrated by the electrochemical characterization of several multiheme cytochromes (10). However, the potential for energy acquisition remains unclear, especially given the relatively small energetic gains from coupling the Mtr pathway to anaerobic terminal electron acceptors (16). Coupling cathode oxidation with oxygen reduction has been observed previously in other organisms (13, 14), though it has never been specifically reported in *S. oneidensis* MR-1. Thermodynamically, oxygen allows for a high relative energy gain compared with that of many terminal electron acceptors. However, it is unknown whether *S. oneidensis* MR-1 cells are able to couple electrons from an extracellular source to oxygen reduction in a way that allows the generation of a proton motive force (PMF). Given the highly enriched cytochrome network in *Shewanella*, it is plausible that nonspecific reduction reactions occur between cytochromes and oxygen, resulting in a catalytic reduction of oxygen without concomitant proton pumping. Conversely, reversing the EET pathway may result in electrons entering the cellular quinone pool and/or interacting with one or more of the inner membrane cytochromes in a way that allow electrons to flow into existing electron transport chain components and, ultimately, into one of the three terminal oxygen-reducing cytochrome reductases (i.e., into *cbb*_3_, *aa*_3_, or *bd*) (18).

To better understand energy acquisition by *S. oneidensis* MR-1 under cathodic conditions, we used an electrode to impose electron-donating redox potentials in an aerobic environment lacking exogenous organic carbon sources. Under these conditions, we set out to understand (i) whether or not electrons from a cathode that enter *S. oneidensis* MR-1 can be utilized for acquisition of cellular energy and (ii) what pathways are involved in electron flow from a cathode to oxygen. Understanding the physiology behind these biologically mediated cathodic processes may allow us to optimize and/or utilize microbes for various microbe-electrode applications, such as electrosynthesis, as well as to better understand microbial physiology under a variety of redox conditions.

## RESULTS

### Electrons flow from a cathode to the *S. oneidensis* MR-1 cellular electron transport chain.

Oxygen-reducing cathode conditions were investigated in three-electrode electrochemical cells using working electrodes covered with a monolayer biofilm on indium-tin-doped oxide (ITO)-coated glass. Significantly more cathodic current was generated than under control conditions (abiotic/cell-free medium and killed-cell biomass) at voltages as high as −203 mV compared to the voltage of a standard hydrogen electrode (SHE), and this effect was linked to the presence of oxygen in *S. oneidensis* MR-1 monolayer cathode biofilms (see Fig. S1 in the supplemental material). Negative currents were observed in *S. oneidensis* MR-1 at potentials of up to −103 mV versus the SHE (data not shown); however, the majority of experiments were run at −303 mV to maintain a high signal-to-noise ratio over a range of experimental conditions. Notably, the redox potential of activities in these biofilms are several hundred millivolts higher than the experimentally observed redox potentials for electrochemical hydrogen production under these conditions (−550 to −600 mV versus the voltage of the SHE) and the theoretical standard redox potential for hydrogen at pH 7 (*E*°′ = −414 mV versus the voltage of the SHE). Additionally, although *S. oneidensis* MR-1 contains hydrogenases and can grow anaerobically on hydrogen, these hydrogenases are not expressed and are thought to be inactive under oxic conditions (19, 20). Comparing the rates of electron flow between anodic (+397 mV, anaerobic, 10 mM lactate) and cathodic (−303 mV, aerobic, no exogenous electron donor) conditions for the same monolayer biofilms, cathodic conditions yielded on average 23.7 ± 5 (*n =* 4) times more current consumption than production. The possibility of these cathode-derived electrons entering the cellular electron transport chain (ETC) was investigated in this system using the combination of a redox active dye (RedoxSensor Green [RSG]) and ETC inhibitors. RSG is a lipid-soluble redox-active dye, previously shown to fluoresce in actively respiring aerobic and anaerobic microbial cells (21–23).

10.1128/mBio.02203-17.1 FIG S1  Current consumption linked to the presence of living cells and oxygen in MR-1 cathodic biofilms. Electrodes were poised at −203 mV versus the voltage of the SHE in a representative (one of six experiments) *Shewanella oneidensis* MR-1 monolayer biofilm (A), a killed MR-1 control (B), and a cell-free abiotic control. Conditions were varied, as indicated by arrows between purges with air or pure nitrogen gas. Download FIG S1, EPS file, 1.7 MB. Copyright © 2018 Rowe et al. 2018 Rowe et al. This content is distributed under the terms of the Creative Commons Attribution 4.0 International license.

**FIG 2:**
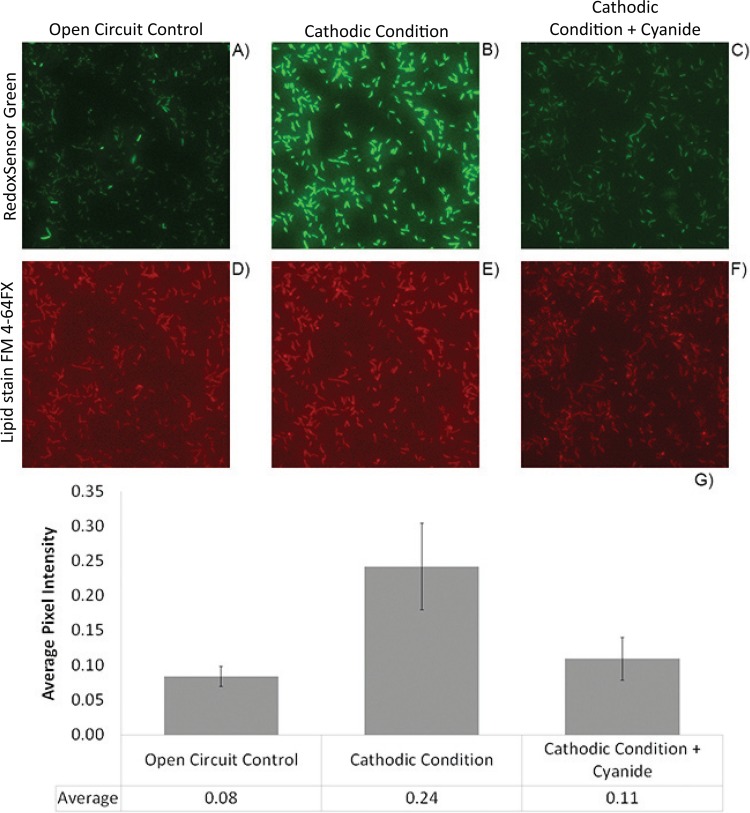
RedoxSensor Green highlights electron flow through the cellular electron transport chain under cathodic conditions. (A to F) Representative images of MR-1 cells attached to ITO-coated glass and treated with RedoxSensor Green (A to C) and the lipid stain FM 4-64FX (D to F) are as described in Materials and Methods. Fluorescence intensities are compared between the control conditions (open circuit) (A and D), cathodic conditions (−303 mV versus the voltage of the SHE) (B and E), and cathodic conditions with an inhibitor of cytochrome *c* oxidase added (−303 mV versus the voltage of the SHE with 5 mM KCN added) (C and F). Average pixel intensity per cell was calculated for approximately 80 images for six time points per condition (average numbers of cells per image, 2,271, 1,904, and 2,234 for the control, cathodic, and inhibition conditions, respectively). Error bars indicate standard deviations in average pixel intensities (per population) per image (80 images were analyzed per experimental condition).

RSG fluoresces green when reduced, and active accumulation of the reduced dye within cells can be linked to respiratory conditions (i.e., active downhill electron flow through the ETC). While the specific oxidoreductases involved in RSG reduction are not known, previous work with *S. oneidensis* MR-1 investigated the effects of ETC inhibitors during aerobic growth on lactate and oxygen (21). Inhibition of RSG activity was seen when an ETC inhibitor was utilized; specifically, it was noted that inhibition of the electron flow at or prior to the terminal oxidase step also interfered with the cellular reduction of RSG (21). This suggests that interaction with a high-potential cytochrome is responsible for RSG reduction in *Shewanella*. Using UV fluorescence microscopy on transparent ITO electrodes, we quantified an increase in RSG fluorescence in *S. oneidensis* MR-1 cells under conditions of applied cathodic potential (−303 mV versus the voltage of the SHE) in the presence of oxygen. Minimal fluorescence was observed under open-circuit conditions. The fluorescence signal seen under cathodic conditions was strongly inhibited by potassium cyanide, a respiration inhibitor (Fig. 2). These observations support the notion that an active cellular electron flow is required for active RSG reduction and accumulation in cells.

**FIG 3:**
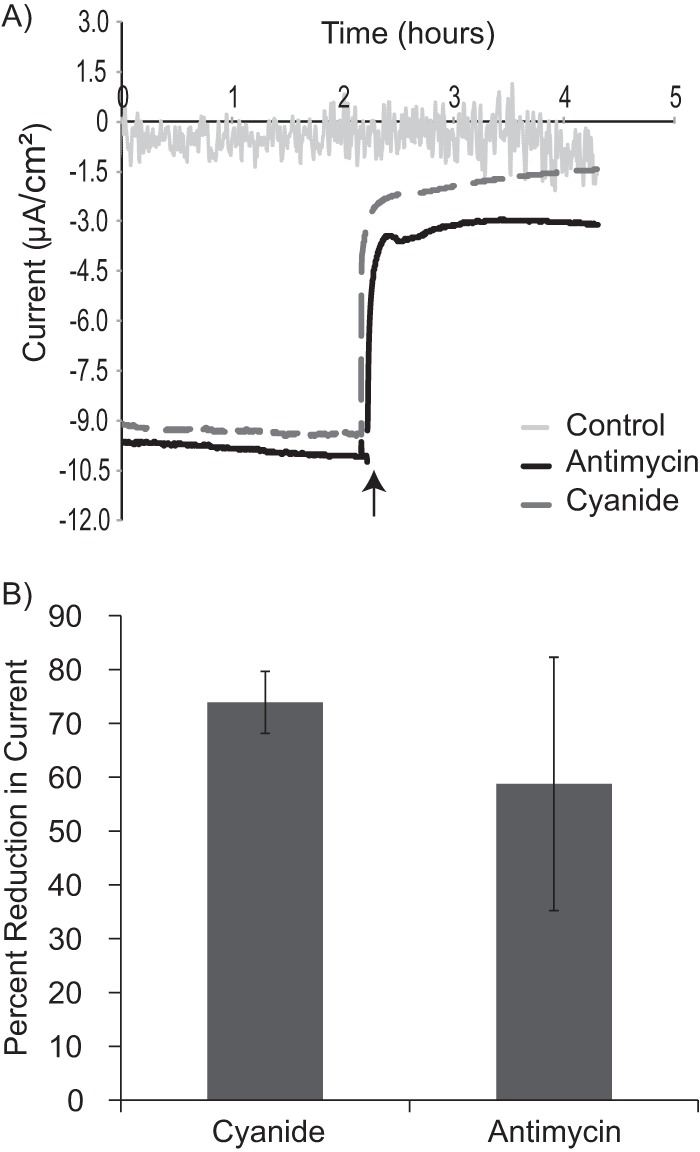
The cathodic current is inhibited by cyanide addition. (A) Sample chronoamperometry plots for MR-1 cells attached to a cathode (results of one of three experiments are shown) and a cell-free control electrode (−303 mV versus the voltage of the SHE), with addition of the electron transport chain inhibitors potassium cyanide (5 mM) and antimycin A (20 µM) (addition is indicated by the arrow), which inhibit cytochrome *c* oxidase and quinone oxidoreductases, respectively. The control sample was treated with both potassium cyanide and antimycin A at the time indicated. (B) The average percentages of reduction in cathodic current ([average cathodic current 1 h preinhibition − the average cathodic current 1 h postinhibition]/average cathodic current 1 h preinhibition) when electron transport chain inhibitors were added are illustrated for 3 reactors. The average total (100%) current for all experiments before inhibitor addition was −6.9 ± 2.7 µA/cm^2^. Error bars represent 1 standard deviation from results of triplicate experiments.

Time-lapse videos from these experiments demonstrate the rapid nature of this process; a marked increase in RSG signal intensity can be observed within 15 min of applying a cathodic potential (videos S1 to S3, available upon request). Additionally, RSG fluorescence significantly decreased within 15 min after addition of potassium cyanide, an inhibitor of cytochrome *c* oxidase (Fig. 2; videos S1 to S3, available upon request). Cathodic current was also mitigated by cyanide addition (between 67 and 78% loss of cathodic current) (Fig. 3), supporting a requirement for terminal cytochrome *c* oxidases to facilitate electron flow from a cathode. Removal of cyanide from a cathode biofilm after a 30-min exposure allowed for recovery of both the cathodic current (68% of the wild-type current was recovered) and RSG fluorescence, suggesting that this effect is due to the reversible inhibition of cytochrome *c* oxidases.

While cyanide is a general cytochrome oxidase inhibitor and several cytochrome oxidases have been shown to pump protons (24), inhibition at quinone proton translocation sites was also tested. Addition of antimycin A (an inhibitor of quinone oxidoreductases) also resulted in a rapid and marked decrease in cathodic current but had no effect on abiotic controls (Fig. 3). Notably, a 60 to 70% loss in cathodic current ([cathodic current − inhibited current]/cathodic current) was observed within the first minute of inhibitor addition (Fig. 3). RSG activity was monitored in cathode biofilms under all inhibitor conditions. While a dissipation of fluorescence can still be observed using antimycin A (videos S4 to S6, available upon request), the quantification of RSG postaddition was made difficult by the autofluorescence of antimycin A. Though antimycin A, as a quinone mimic, may interact nonspecifically with other quinone oxidoreductases, it has been shown to preferentially inhibit the oxidation of ubiquinone by the cytochrome *bc*_1_ complex in mitochondria (25). These results demonstrate that electron flow from a cathode passes through at least one coupling site in the cellular electron transport chain.

### Cellular energy carrier quantification in cathode-oxidizing *S. oneidensis* MR-1 cells.

**FIG 4:**
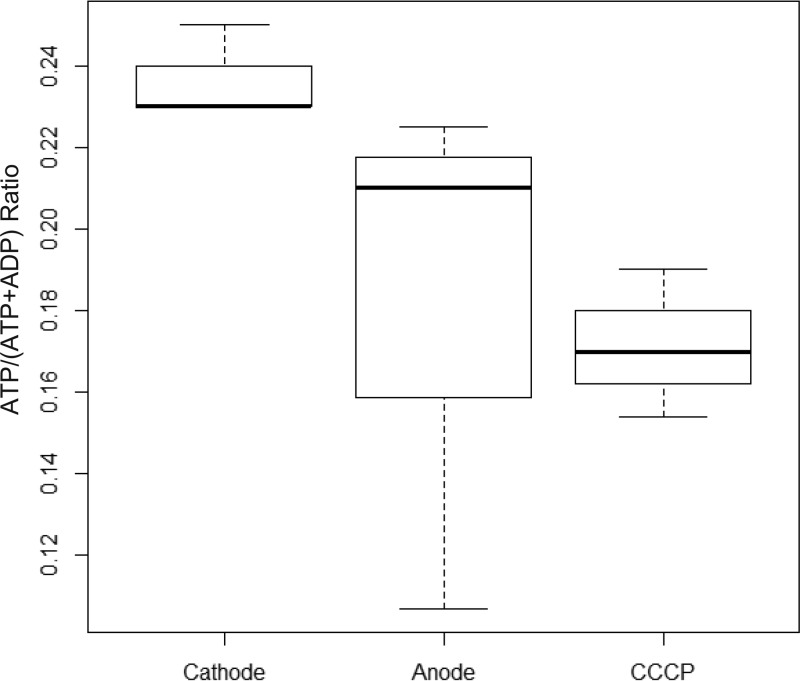
Larger ATP pools were observed under cathodic conditions. ATP levels were normalized to those of combined ATP and ADP levels recovered from MR-1 biofilms maintained under poised electrode conditions (−303 or 197 mV versus the voltage of the SHE) for 24 h. The conditions compared reflect cathodic-current generation with an electrode potential poised at −303 mV (Cathode) (*n* = 9), minimal anodic-current generation with an electrode potential of 197 mV (Anode) (*n* = 9), and cathodic-current generation as described above with a 3-h CCCP treatment prior to ATP recovery (CCCP) (*n* = 9). All treatments were performed under exogenous-carbon-free and aerobic environmental conditions. Box plot reflects the median values (bold black lines), 1 standard deviation around the mean (box), and the data spread (dashed lines).

To further investigate whether a proton gradient is generated under cathodic conditions that might consequently result in ATP generation, we measured pools of ATP and ADP within cathode biofilms. We compared the ATP/(ATP plus ADP) ratios (to normalize for differing overall cellular nucleotide levels) for replicate biofilms exposed to either cathodic conditions (−303 mV versus the voltage of the SHE), cathodic conditions plus treatment with the protonophore uncoupler carbonyl cyanide *m*-chlorophenyl hydrazine (CCCP) for 3 h, and poised potential conditions (197 mV versus the voltage of the SHE) where minimal anodic current flow was observed (average of five replicates, 0.19 ± 0.4 µA). This anodic control utilized environmental conditions mirroring the cathodic condition (no carbon added, oxygen provided, etc.) while altering the potential on the electrode to a level where a minimal reduction current occurs, therefore preventing cathodic electron flow. This control accounts for background heterotrophy and/or cellular energy obtained from storage products in this carbon-free system. One caveat is the difficulty in controlling for variation in the physiological responses to changes in potential, including possible increased carbon/electron equivalents added to this system from decaying cell biomass or hydrogen produced on the counterelectrode. Nonetheless, the ATP/(ADP plus ATP) ratio was significantly higher under cathodic conditions than under either control condition (Fig. 4). While we did not quantify AMP levels, the ATP(ATP plus ADP) ratios also supported an increase in the energy charge state (26) of the cells under cathodic conditions.

Statistically significant cell loss was observed in open-circuit controls (Fig. S8), likely due to the lack of energy input required to maintain cell biomass on the electrode. Because of this, ATP levels fell below that assay detection limit. Unlike with the open-circuit controls, cell biomass does not statistically change throughout the course of the experiments when poised electrodes have been used (2.2 × 10^7^ ± 5 × 10^6^ and 2.3 × 10^7^ ± 1.2 × 10^7^ cells per biofilm for cathodes and minimal anodes, respectively). The per-cell ATP values estimated in this work fall between 0.13 and 0.68 fmol of ATP per cell (7.8 × 10^7^ to 4.1 × 10^8^ ATP molecules per cell). Though cellular ATP levels can vary across microbes, as can growth rates (shown to range 6 orders of magnitude across taxa) (27), the per-cell ATP levels observed in this work were similar to those of environmentally sampled *Escherichia coli* cells (0.18 to 0.25 fmol per cell) (27, 28).

The observed difference in ATP/(ATP plus ADP) ratios between wild-type *S. oneidensis* MR-1 cathodic biofilms and *S. oneidensis* MR-1 cathodic biofilms treated for 3 h with CCCP (Fig. 4) supports the notion that *S. oneidensis* MR-1 generates a proton gradient during cathode oxidation, as ATP levels are lower in the presence of an uncoupler. While the differences observed between cathodic experiments with and without the uncoupler are small, they are significant on a per-cell basis, as they equate to a minimum difference of 4 million ATP molecules. These 4 million molecules, assuming a steady-state ATP concentration under cathodic conditions, represent the net consumption of ATP over a 3-h period (equaling the amount produced over the time frame of CCCP addition to maintain steady-state levels). However, this may capture only a small fraction of the per-cell ATP flux (the total ATP production over time accounts for losses due to consumption). The amount of current required to generate 4 million ATP molecules in a cell is 0.19 to 0.05 fA over a 3-h period (assuming 1 to 4 protons pumped per electron and 3.3 protons required per ATP). Though this is the lowest conservative estimate of ATP being generated in a 3-h period, these values represent 0.2 to 0.01% of the range of per-cell currents observed (50 to 100 fA) over this time frame. This estimate does not account for the total potential ATP turnover within cells and/or other sinks for PMF under these conditions, both of which might be significant given the unaccounted-for PMF. For example, depending on the degree of proton accumulation, this PMF might also aid in reverse electron flow (i.e., electron flow from the generally higher potential quinone pool to oxidized cellular electron carriers), resulting in the generation of cellular reducing equivalents (i.e., NADH) from the cathodic electron flow (model illustrated in Fig. 1B).

To directly test whether cathodic electron flow can be converted to cellular reducing power in the form of NAD(P)H and/or FMNH_2_, we used the bacterial luciferase enzyme, inserted downstream of the *glmS* gene into a neutral site in the bacterial genome via transposition with the *lux* operon (*luxCDABE*) (29) as a real-time *in vivo* marker for the cellular redox pool. The Lux enzyme system performs a well-characterized cytoplasmic process that generates light using an oxygen molecule, reduced flavin mononucleotide (FMNH_2_), and an activated (via NADPH and ATP) aldehyde functional group (30). Though there are multiple factors that influence light production, the cellular redox state (overall ratio of reduced to oxidized cellular electron carriers) has been shown to be proportional to the amount of light produced by the cell (31–33). In these experiments, expression of the *lux* operon is constitutively driven by the P1 promoter, resulting in consistent light production under aerobic growth conditions. The variation of enzyme levels, depending on the growth history of the cells, made it difficult to compare levels of light production quantitatively across different experiments where the energy investment in protein production varied. However, comparing across cell populations with similar growth histories and maintaining nonlimiting oxygen and aldehyde concentrations for the Lux reaction, we could correlate lactate concentration (which in turn affects respiration rate, electron flow, and the redox pool) to light production across normalized cell populations (Fig. S2). This provides support to the possibility of the Lux reaction yielding information about the cellular energy state, despite natural variation across populations.

10.1128/mBio.02203-17.2 FIG S2  Light production in the *SO-lux* mutant varies with lactate concentration and the growth history of cells. Light production of MR-1 cells grown in defined medium (DM) with lactate, normalized to an optical density (OD) at 600 nm of 0.2, and resuspended in fresh DM with various concentrations of lactate, specifically, 7 mM, 0.7 mM, and 0 mM (no additional lactate added; >0.07 mM). Results from four different biological replicates with DM-lactate-grown cell cultures are illustrated. Average light production over a 20-min period after density normalization in fresh medium (the maximum growth rate in this medium under these conditions is >1 h). Download FIG S2, EPS file, 1.3 MB. Copyright © 2018 Rowe et al. 2018 Rowe et al. This content is distributed under the terms of the Creative Commons Attribution 4.0 International license.

**FIG 5:**
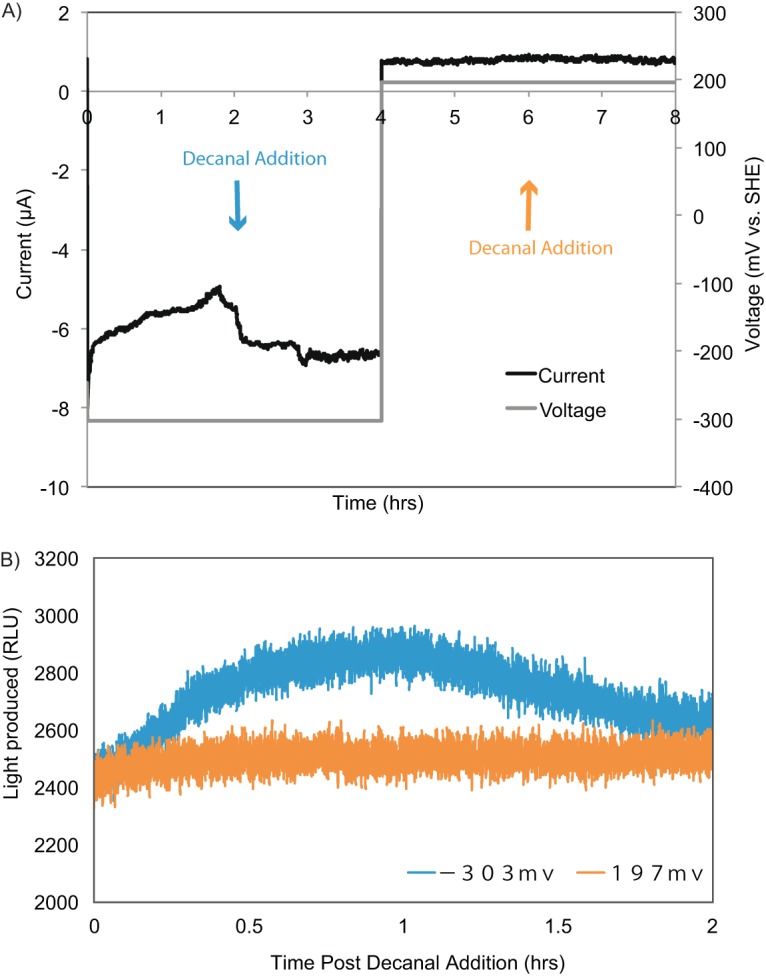
Luciferase light production is significantly higher under cathodic conditions than under anodic conditions for the same biofilm. (A and B) Representative demonstrations (one of four) of the *SO-lux* mutant electrode biofilm current (A) and light production for an electrode poised at a cathodic-current-generating redox potential (−303 mV versus the voltage of the SHE, 0 to 4 h) and then switched to a noncathodic redox potential (197 mV the voltage of the SHE, 4 to 8 h), where minimal anodic current was observed (B). Decanal was added at a concentration of 0.002% at 2 h into each poised potential incubation (as indicated by the colored arrows). (B) Light production was quantified by a photon-multiplying tube and is presented in relative light units (RLU) for the 2-h period after decanal addition, which corresponds to the 2-h period following the blue and orange arrows shown in panel A.

Light production was limited for aldehyde under cathodic conditions (no exogenous carbon), as demonstrated by the rapid increase in light production when 0.002% decanal was provided (a modest initial increase likely due to aldehyde diffusion across the cell membrane), peaking 0.5 h after decanal addition (Fig. 5). Increased light production does not appear to be based on decanal conversion to reducing power, as no light was observed under the control conditions (minimal anodic current condition), and *S. oneidensis* MR-1 did not grow aerobically with decanal as the sole carbon source. Though it was difficult to compare light intensities across various experiments, the trend of increasing light production with increasing current consumption compared to levels in controls was consistently observed and was independent of the order of poised potential conditions (Fig. S3). We also noted that the magnitude of current generated was positively correlated with light production (Fig. S3). Given the variety of potential sources of reducing equivalents in *S. oneidensis* MR-1 (cellular storage products, endogenous cell decay, etc.), it is difficult to determine whether there is a direct link between cathodic electrons and the cellular reducing pool (total cellular electron carriers) from these data alone. However, the increase in the cellular reducing pool observed in *SO*-*lux* strains (strains in which the *lux* operon was inserted into the *S. oneidensis* MR-1 genome via transposition at a neutral site via a mini-Tn*7*–*luxCDABE*–*tp* cassette), along with the likelihood of PMF generation from the cathodic electron flow, supports the possibility that reverse electron flow operates in *S. oneidensis* MR-1 cells under cathodic conditions.

10.1128/mBio.02203-17.3 FIG S3  Light production in the *SO-lux* mutant increases with a decrease in the poised potential or an increase in electron uptake. Light production generated after decanal addition to a representative *SO-lux* mutant cathode biofilm poised sequentially at 197 mV and −3 mV, followed by −303 mV (versus the voltage of the SHE). Decanal (not metabolized my MR-1) addition prevents aldehyde limitation for the luciferase reaction, such that reducing equivalents are the remaining limiting factor for this reaction. Cathodic current increased as poised potential decreased, suggesting a corresponding increase in cellular redox pools. Biofilms were allowed to equilibrate 2 h prior to decanal addition. Decanal addition is indicated by a colored arrow for each potential. Download FIG S3, EPS file, 2.5 MB. Copyright © 2018 Rowe et al. 2018 Rowe et al. This content is distributed under the terms of the Creative Commons Attribution 4.0 International license.

### Reverse electron flow enhances the cellular reducing pool in *S. oneidensis* MR-1.

**FIG 6:**
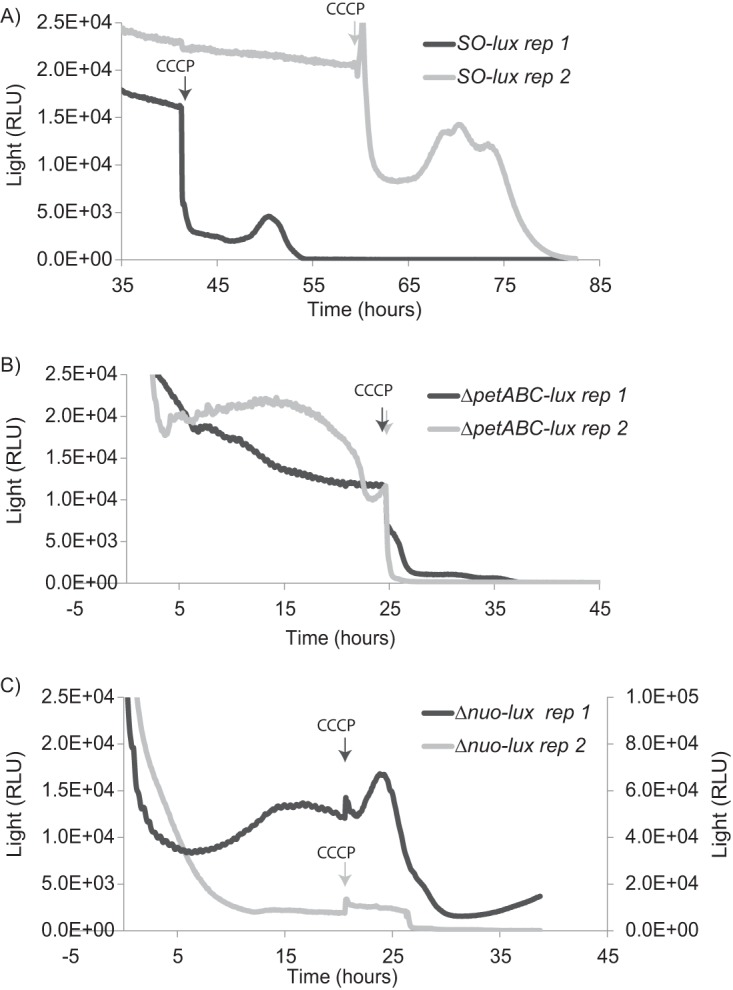
Light production is inhibited by CCCP addition, unless the complex I gene is deleted. (A to C) Light was quantified in cathode biofilms poised at −303 mV (versus the voltage of the SHE) for two (of three) replicates (rep 1 and 2) of the following MR-1 strains amended with the *lux* operon: wild-type MR-1 (*SO*-*lux*) (A), the *bc*_1_ complex mutant (Δ*petABC-lux*) (B), and the complex I mutant (Δ*nuo-lux* mutant) (C). After at least 20 h under cathodic conditions and at the time points indicated by arrows on each plot, the protonophore uncoupler CCCP was added to each reactor. Light was measured via a photon multiplier tube and is presented in relative light units (RLU). Plots depict the 45- to 50-h period around CCCP addition. Replicate 1 with the Δ*nuo-lux* mutant demonstrated 2-fold-higher light intensities, and its results are therefore plotted on a larger light intensity scale (right axis in panel C).

Reverse electron flow involves utilizing a proton gradient to drive electrons from a higher potential state (i.e., quinone pool) to a lower potential state (i.e., NAD^+^) in the cellular electron transport chain (34, 35). Under conditions of reverse electron flow, generation of NADH can be inhibited by a protonophore uncoupler (36). To determine whether cathodic electron flow resulted in an enhanced redox pool through reverse electron flow, the protonophore uncoupler CCCP was used in combination with the *SO*-*lux* strain to determine whether collapse of the inner membrane proton gradient affected light generation via the luciferase enzyme, with light utilized as an intracellular marker for the cellular redox pool. Addition of CCCP to the *SO-lux* strain showed a marked decrease (by nearly an order of magnitude) in light production (Fig. 6A), though no statistically significant effect was observed on the current (Fig. S4). The inhibitory effects of CCCP on light production were mitigated by addition of lactate under otherwise-identical cathodic conditions (Fig. S5). It is difficult to distinguish between the effects of ATP and NAD(P)H in these *SO-lux* strain experiments, as both NAD(P)H and ATP are (i) required for the luciferase reaction, (ii) likely linked to proton motive force (either via reverse electron flow or ATPase activity) and are therefore affected by CCCP addition, and (iii) products of lactate metabolism (ATP via substrate-level phosphorylation and NADH via lactate dehydrogenase activity) independently of PMF, which might explain lactate mitigating the effects of CCCP. This makes it difficult to link the effects of CCCP to the reverse electron flow using these wild-type experiments alone. Because of this, *SO-lux* strains with various electron transport chain components deleted were also evaluated.

10.1128/mBio.02203-17.4 FIG S4  Small increases or no change in current consumption in MR-1 strains resulted upon the addition of CCCP. Current density data correspond to light quantification experiments shown in Fig. 6. Arrows indicate the time of CCCP addition for the *SO-lux* mutant (A), the Δ*petABC*-*lux* mutant (B), and the Δ*nuo*-*lux* mutant (C) MR-1 strains. Electrodes were poised at −303 mV (versus the voltage of the SHE) for all experiments. Download FIG S4, EPS file, 10.6 MB. Copyright © 2018 Rowe et al. 2018 Rowe et al. This content is distributed under the terms of the Creative Commons Attribution 4.0 International license.

10.1128/mBio.02203-17.5 FIG S5  Light production is unaffected by CCCP in the *SO-lux* mutant if an electron donor (lactate) is provided under cathodic conditions (A), under electron donor-limited noncathodic conditions (B), or when the complex I enzyme is inhibited. Light was quantified in *SO*-*lux* cathode biofilms poised at −303 mV (A and C) and 197 mV (B) versus the voltage of the SHE. All cathode experiments were run in carbon-free defined media (CF-DM) under aerobic conditions. Lactate was added to compete with the reverse electron flow for the generation of NADH (A), and piericidin A (PA) was added to inhibit complex I and in turn to inhibit the reverse electron flow (B). Arrows indicate addition of the protonophore uncoupler CCCP (~17 h into experiments). Light was measured via a photon multiplier tube and is presented in relative light units (RLU). Download FIG S5, EPS file, 24.9 MB. Copyright © 2018 Rowe et al. 2018 Rowe et al. This content is distributed under the terms of the Creative Commons Attribution 4.0 International license.

A similar decline in light production was observed with the *bc*_1_ complex mutant of *S. oneidensis* containing the *lux* operon (Δ*petABC-lux* strain) when CCCP was added (Fig. 6B). The *bc*_1_ complex is not essential to the aerobic respiratory electron transport chain (37), as *S. oneidensis* MR-1 has multiple ETC routes to reduce oxygen (18, 38). However, the PetABC complex has the potential to maximize PMF generation under aerobic conditions (i.e., in the Q cycle versus the Q loop) (36). Additionally, the observed loss of light production upon CCCP addition suggests that PetABC is not involved in mediating electron flow to the cellular redox pool in *S. oneidensis* MR-1, as has been suggested for other organisms (39, 40). Unlike with other mutants, in the Δ*nuo-lux* strain (complex I deletion in *S. oneidensis* is amended with *lux*), CCCP addition did not result in a decline in light production within the first 15 min of addition (Fig. 6C). Complex I is a reversible proton-pumping NADH/ubiquinone-oxidoreductase (41, 42) that has been implicated in reverse electron flow in other organisms (34, 35, 43, 44). In the Δ*nuo-lux* strain, steady light production was observed for a >5-h period until ultimately declining, consistent with the ultimate decline in cell health resulting from CCCP addition. The observation that, in the absence of complex I, PMF does not have the same effect on cellular light production implicates reverse electron flow in *S. oneidensis* MR-1. The most probable cause for this phenomenon in the Δ*nuo-lux* strain compared to the wild type is the inability to couple PMF and NADH reduction, which would explain the lack of change in the cellular reducing pool (or light production) upon CCCP addition. This result could be recreated in the wild-type *SO-lux* strain when the complex I-specific inhibitor piericidin A was added to a cathodic biofilm. Piericidin A addition prevented the decrease in light production observed in the wild-type cells alone (Fig. S5). This further supports a role for complex I in generating reducing equivalents via a reverse electron flow under cathodic conditions in *S. oneidensis* MR-1.

CCCP also did not affect light production under the control condition where a minimal oxidizing potential was poised and a minimal anodic current was observed (averages, 23 ± 1 and 34 ± 3 nA) (Fig. S5). Though it is difficult to exclude the possibility that ATP is involved in the effects on light production seen upon CCCP addition, in order for ATP levels to drive the results seen with the Δ*nuo-lux* strain with piericidin A and the minimal-anodic-current controls, the standing ATP pools in these experiments would have to be significantly larger than in wild-type experiments. There is no obvious mechanism that would support this phenomenon (enhanced ATP pools) in all three scenarios. This supports the likelihood of reverse electron flow under cathodic conditions being the major driver of these observations, which from the cumulative luciferase experiments demonstrate dependence on (i) cathodic electron flow, (ii) an oxidized redox pool and/or electron donor-limited conditions, and (iii) the presence of a reversible proton-translocating NADH/ubiquinone oxidoreductase for reverse electron transport to occur.

### Cathode oxidation results from a reversal of multiple extracellular electron transport routes in *S. oneidensis* MR-1.

To assess the route of electron flow into cells under cathodic conditions, we utilized a set of gene deletion mutants of various extracellular electron transport pathways, as well as inner membrane electron transport chain mutants (Table 1; illustrated in Fig. 1). Of the mutants tested, the mutant deficient in all three of the terminal cytochrome oxidases (Δ*cox-*all mutant) demonstrated the greatest percentage decrease in current consumption, though the individual cytochrome oxidase mutants displayed near wild-type cathodic-current levels (Table 1; Fig. S6). Deletion of all five outer membrane multiheme cytochromes (Δ*omc-*all mutant) also significantly decreased current consumption; it was only slightly greater than that of the Δ*mtrC* Δ*omcA* double mutant (Table 1; Fig. S6). However, minimal cathodic currents were observed in the majority of other cytochrome mutants tested, including a variety of periplasmic electron carriers. Surprisingly, this included proteins like DmsE, a periplasmic decaheme cytochrome shown to be associated with dimethyl sulfoxide (DMSO) reduction (45). Some cathodic-current generation could be recovered when a copy of the *dmsE* gene behind the ribosomal binding site of *mtrA* was introduced into the Δ*dmsE* mutant in *trans*. Coupled with the observation that other *dms* deletion mutants (*dmsB* and Δ*dms*-all mutants) significantly decrease cathodic-current generation, there is the potential that multiple extracellular electron transport pathways run in parallel and/or cooperatively during cathode oxidation. It is difficult, however, to distinguish the hypothesis of parallel pathways of cathodic electron flow from the possibility of differential gene expression affecting cathodic-current generation among the various mutants.

10.1128/mBio.02203-17.6 FIG S6  Deletion of heme-containing proteins in MR-1 affects current consumption. Cathodic currents per nanogram of protein are shown for various MR-1 gene deletion mutants (listed in Table 1) for electrochemical cell biofilms poised at −303 mV versus the voltage of the SHE. Currents were average over 24 h. Error bars are standard deviations of results from replicate reactors (*n* ≥ 3). Download FIG S6, EPS file, 1.2 MB. Copyright © 2018 Rowe et al. 2018 Rowe et al. This content is distributed under the terms of the Creative Commons Attribution 4.0 International license.

**TABLE 1:** *Shewanella oneidensis* MR-1 strains used in this study

Strain	Gene deletion(s)	Reference or source^a^	Description	% reduction in normalized cathodic current compared to that of the wild type^b^
MR-1 wild type		55		
Δ*mtrC* Δ*omcA* mutant	SO1778, SO1779	68*	Outer membrane decaheme cytochrome gene deletions	88.2 ± 6.7
Δ*omc*-all mutant	SO1778–SO1782, SO2931, SO1659	69*	Deletion of all outer membrane multiheme cytochrome gene homologs	95.7 ± 3.2
Δ*cymA* mutant	SO4591	68*	Tetraheme cytochrome *c* quinone oxidase gene deletion	85.2 ± 0.1
ΔPEC mutant	SO1777, SO1782, SO1427, SO4360, SO2277	65*	Deletion of genes for periplasmic electron carriers: MtrA, MrtD, DmsE, SO4360, CctA (small periplasmic tetraheme cytochrome)	82 ± 1.3
Δ*mtrA* mutant	SO1777	68*	Periplasmic decaheme cytochrome gene deletion	72.3 ± 4.5
Δ*fccA* mutant	SO0970	68	Fumarate reductase, periplasmic tetraheme flavochrome gene deletion	4.4 ± 11.2
Δ*dmsE* mutant	SO1427	68	Gene deletion of MtrA homolog involved in DMSO reduction	92.4 ± 0.3
Δ*dmsE*::*p*(*mrtA*_*rb*_)*dmsE* mutant^c^	SO1427	This work	*dmsE gene* deletion mutant containing the plasmid *dmsE* gene 38 bp upstream of the *mtrA* sequence in pBBR1MCS-2 (65)	57.6 ± 3.8
Δ*dmsB* mutant	SO1430	45*	Lacking the iron-sulfur cluster subunit of the Dms operon	93.5 ± 1.2
Δ*dms-*all mutant	SO1427–SO1432	This work	Lacking the DMSO reductase operon (*dmsEFABGH*)	91.8 ± 0.3
Δ*petABC* mutant	SO0608–SO0610	This work	Lacking the cytochrome *bc*_1_ complex genes	86.3 ± 1.8
Δ*nuo* mutant	SO1018 (partial), SO1019–SO1017	This work	Full-gene deletion of *nuoE*, a portion of *nuoF* (5′) from the NADH-oxidizing N module, and partial deletion of *nuoC* and *nuoD* (3′) from the iron sulfur-containing Q module	95.3 ± 4.4
Δ*ccoO* mutant	SO2363	68	Deletion of the gene for cytochrome *c* oxidase, *cbb*_*3*_ type, subunit II	40.7 ± 2
Δ*cox*-all mutant	SO2361–SO2364, SO3285–SO3286, SO4606–SO4609	This work	Lacking all the terminal cytochrome *c* oxidase genes, including *cbb*_3_, *bd* (low oxygen concentration), and *aa*_3_	96.5 ± 1.2
*SO*-*lux* mutant		This work	Mini-Tn*7*–*lux*(*CDABE*)*–tp* cassette inserted into a neutral site downstream of the *glmS* gene (29) in the wild-type background	NC
Δ*petABC*-*lux* mutant	SO0608–SO0610	This work	Mini-Tn*7*–*lux*(*CDABE*)*–tp* cassette inserted downstream of the *glmS* gene (29) in the Δ*petABC* mutant background	NC
Δ*nuo*-*lux* mutant	SO1018 (partial), SO1019–SO1017	This work	Mini-Tn*7*–*lux*(*CDABE*)*–tp* cassette inserted downstream of the *glmS* gene (29) in the Δ*nuo* mutant background	NC

^a^ An asterisk^ ^indicates that mutant complementation was performed in the indicated reference.

^b^ Shown are the percentages of reduction in the normalized cathodic current observed (Fig. S6) in gene deletion mutants compared to the cathodic current of the wild type (values were not calculated [NC] for *lux* insertion mutants).

^c^ Promoter of gene altered to contain the ribosomal binding (*rb*) site of the *mrtA* gene (SO1777).

**FIG 7:**
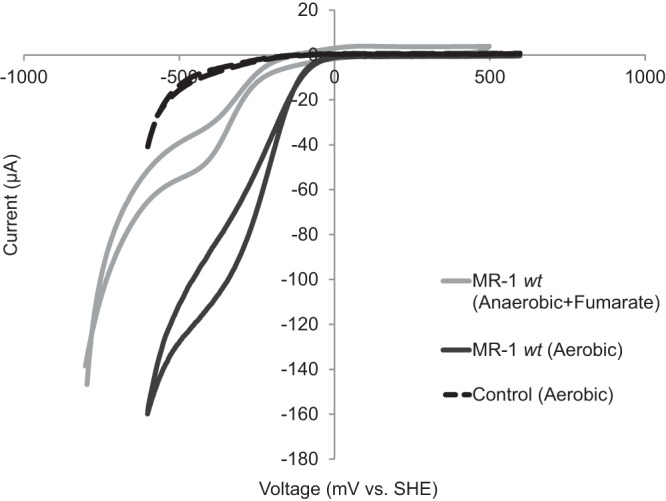
Cathodic electron uptake in MR-1 cells shifts to more positive potentials under oxic conditions than under anoxic conditions. Shown are cyclic voltammetry curves taken for an MR-1 cathodic biofilm poised at −303 mV (versus the voltage of the SHE) incubated under aerobic conditions with oxygen as a terminal electron acceptor and under anaerobic conditions with fumarate as a terminal electron acceptor (17). CV results of one of three experiments are also shown for a cell-free abiotic control under aerobic conditions (dashed line). Scans were run at 5 mV/s under aerobic conditions and 10 mV/s under anaerobic conditions. *wt*, wild type.

Cyclic voltammetry (CV) was performed on all mutant strains utilized in these experiments. The only two mutants to demonstrate CV profiles for aerobic cathodic electron flow similar to that of the wild type (Fig. 7) were the Δ*fccA* and Δ*ccoO* mutants (data not shown). The other mutants tested showed a significantly decreased onset potential and/or magnitude of cathodic electron flow that was only slightly enhanced from those of controls (data not shown), consistent with the observed decrease in current generated during chronoamperometry experiments for these mutants. Interestingly, a 180- to 200-mV increase in onset potential was observed when we compared *S. oneidensis* MR-1 aerobic/oxygen-reducing cathodic biofilms and *S. oneidensis* MR-1 anaerobic/fumarate-reducing cathodic biofilms (Fig. 7). Under aerobic conditions, we observed a 0- to 20-mV cathodic-current onset potential versus that of the SHE, whereas under anaerobic conditions with fumarate, we observed an onset potential of −160 to −180 mV versus that of the SHE (Fig. 7); these match the corresponding potentials proposed for the outer membrane cytochromes with unbound and bound flavins, respectively (46). These observed redox potentials further support the role of outer membrane cytochromes in cathode oxidation, though it does not exclude the possible activities of other pathways with similar oxidation reduction potentials. In addition, these results also support the requirement for reverse electron flow to generate cellular reducing equivalents, such as NADH from NAD^+^, given the 300 mV more negative redox potential observed between the midpoint potential of electron uptake in *Shewanella* (0 to 20 mV versus the voltage of the SHE) and NADH (E°′ [pH 7] = −320 mV versus the voltage of the SHE) (47).

### Implications for cathode oxidation in minimizing cellular decay.

As expected, cell growth was not observed over a 5-day period with cathodic conditions imposed (Fig. S7A). However, cell decay (loss of cell biomass through death and lysis) was also not observed under cathodic conditions. Open-circuit conditions demonstrated a statistically significant loss in surface-attached cell biomass over the same experimental period (an approximately 17% cell loss per day) (Fig. S7). This survival stands in sharp contrast to the cellular decay rate of planktonic cells under comparable conditions (with the identical medium and a similar starting optical density [OD]), where a 48% loss of biomass is expected over the same period (Fig. S7). While it is difficult to distinguish cell detachment from cell decay under biofilm conditions, over a 5-day period, the loss of cell biomass observed under the minimal-anodic-current condition is consistent with the amount of cell loss observed under open-circuit conditions (an ~4-day half-life) though not statistically significant within a 24-h period (Fig. S7). These observations add further support to the capacity for cellular energy acquisition under cathode-oxidizing conditions and imply that this energy may be harnessed to provide cell maintenance to stave off cell death and decay and/or maintain cellular attachment in a biofilm.

10.1128/mBio.02203-17.7 FIG S7  Minimal cell decay was observed under cathodic conditions compared to that of controls. (A) Direct cell counts from electrode biofilms (*n =* 4) at days 0, 1, and 5 (the time course for all experiments) are shown. (B) Cell decay for planktonic cell populations (*n =* 3) at the typical starting cell density of planktonic cells in DM minus carbon (see Materials and Methods) and cell loss (*n =* 4) from attached cell biomass during open-circuit control experiments. Decay rates of 0.48 per h and 0.17 per day are predicted for planktonic and open-circuit attached cells, respectively, corresponding to 26- and 105-h half-lives. Download FIG S7, EPS file, 2.4 MB. Copyright © 2018 Rowe et al. 2018 Rowe et al. This content is distributed under the terms of the Creative Commons Attribution 4.0 International license.

## DISCUSSION

This report describes evidence of cellular energy acquisition in *S. oneidensis* MR-1 from cathode oxidation coupled to oxygen reduction. The periplasm and outer membrane of *S. oneidensis* MR-1 are enriched in redox-active cytochromes, raising the possibility that electrons from an exterior cathode are routed along this redox network to a terminal oxidant in a biocatalytic process, rather than that they interact with inner membrane components that would, via generation of an electrochemical gradient, allow cells to acquire energy. This work combines a variety of genetic, biochemical, and electrochemical investigations to understand both the interactions of cathodic electrons with cellular components and the physiological consequences of these interactions.

Though a model for electron flow was developed for *S. oneidensis* MR-1 anaerobic-cathode oxidation using fumarate as an electron acceptor (16, 17), much less was known about electron flow under cathodic conditions with oxygen used as a terminal electron acceptor. Analysis via cyclic voltammetry suggests a distinct redox potential difference in the routes of entry from *S. oneidensis* MR-1 cells under aerobic conditions and anaerobic conditions (Fig. 7). It should be noted that this redox potential is higher than the standard redox potentials for both NAD(P)H and hydrogen at pH 7. Our results are consistent with cathodic activity under aerobic conditions being mediated by a *c*-type cytochrome without a bound flavin, as the redox potentials that we observed are in line with those reported for Mtr proteins lacking flavins (10, 46). This result is consistent with the proposed model that flavin binding is prevented by a disulfide bond formed under aerobic conditions in MtrC, OmcA, and MtrF (48).

While these outer membrane multiheme cytochromes likely play an essential role in cathode oxidation, our data suggest that the canonical *S. oneidensis* MR-1 cytochromes (MtrC and homologs) are not the only cytochromes important for cathodic function (Table 1). Although the use of gene deletion mutants suggests several possible pathways, the degree of decrease in cathode-oxidizing activity in nonredundant or parallel pathways suggest that either (i) the architecture of the cytochrome network is in some way cooperative and/or plays an important functional role in the rate of cathodic electron flow or (ii) differential levels of gene expression across the deletion mutants tested are responsible for the altered cathode oxidation activity. Because of this, the explicit route(s) of electron flow from the cathode remain unclear; however, the interaction of cathodic electrons with the cellular electron transport chain, specifically in protein components that interact with protons (i.e., the *bc*_1_ complex and complex I), is supported by this work (Table 1). Combined with evidence from electron transport chain inhibitor studies (Fig. 2 and 3) and ATP quantification (Fig. 4), these data strongly suggest the generation of proton motive force and subsequent utilization for cellular processes under aerobic and cathodic conditions.

The route of electron flow determined under anaerobic conditions also involves reversal of the Mtr pathway, but energy acquisition under these conditions was not determined (16). The relatively small redox potential difference between an electron in the Mtr pathway and fumarate, as well as the potential for direct interactions between these proteins (8), makes it unlikely that these redox couples drive proton pumping; the standard redox potentials are reported to be lower than those of known coupling sites which utilize >180 mV to translocate a proton (36). Conversely, the energetics for aerobic-cathode conditions, owing to the greater reduction potential of oxygen, is more thermodynamically favorable, which likely results in different physiological consequences between aerobic- and anaerobic-cathode processes, namely, the ability of electrons to enter the aerobic electron transport chain.

The magnitude of PMF accumulation under aerobic-cathode conditions is likely not only linked to the production of ATP. This work presents evidence for the generation of cellular reducing equivalents [i.e., NAD(P)H and FMN] during cathodic conditions (Fig. 5). Experiments utilizing a luciferase bioassay, protonophore uncouplers, and mutants in the aerobic cellular electron transport chain suggest that reverse electron flow (the reduction of lower potential electron carriers utilizing PMF as an energetic driving force) can be utilized by *S. oneidensis* MR-1 to generate reduced cytoplasmic electron carriers under cathodic conditions (Fig. 6). Light production via luciferase has been shown to be directly dependent on FMNH_2_ levels and indirectly on NAD(P)H levels and ATP (30). NAD(P)H, in particular, is required for the activation of the aldehyde moiety (LuxCE) (30) and can be used to regenerate FMNH_2_ that is used directly, a process catalyzed by endogenous cellular enzymes (31, 49–51). While the luciferase reaction is not a quantitative marker of NAD(P)H levels specifically, the effect of CCCP on light production under a variety of scenarios supports the connectivity between cellular reducing equivalents and PMF via reverse electron flow.

The main observations that led to this hypothesis are that under NADH-limiting conditions (i.e., electron donor limitation), in the presence of NADH-ubiquinone oxidoreductase complex I (a reversible enzyme that has been shown to generate NADH using PMF), and with cathodic electron flow, the addition of a protonophore uncoupler results in a rapid decrease (within 15 min) in light production, likely reflecting the loss of NADH generation from reverse electron flow. Subsequently, the lack of complex I, addition of a carbon source, or lack of cathodic electron flow does not result in a similar collapse in the luciferase reaction. Reverse electron flow of electrons from a cathode to NADH mediated by complex I in the presence of predominantly oxidized cellular electron carriers is the model that accounts for these experimental observations. Other hypotheses may account for some of these observations. The collapse in ATP production that occurs due to the loss of PMF might also inhibit the luciferase reaction; however, it is unclear how or why ATP collapse from CCCP would differentially affect the Δ*nuo-lux* strain and the Δ*petABC-lux* and SO*-lux* strains. Additionally, measurable ATP was observed in the wild-type strain after 3 h of CCCP addition (Fig. 4). Alternatively, under predominantly heterotrophic conditions, uncoupler addition can result in high rates of NADH oxidation due to the amelioration of PMF. This can, in turn, inhibit proton-translocating electron transport chain components. If this mechanism occurs, the collapse of light production upon CCCP addition would likely have been observed in both the lactate-amended experiments and the complex I deletion experiments. Notably, MR-1 has alternate and non-proton-pumping NADH/quinone-oxidoreductases (i.e., *ndh*) (52, 53), which allows for NADH oxidation in the Δ*nuo* background.

Reverse electron flow, as has been described in chemolithoautotrophs, utilizes high-potential electron donors as a sole electron source (34, 35, 54). Implicit for cellular biosynthesis and/or carbon fixation is the conversion of NADH to a variety of cellular electron carriers utilized in anabolism, most commonly NADPH (36). These reducing equivalents are critical for biosynthesis, and the observation of enhanced maintenance of cell biomass (or slower death) under cathodic conditions further supports the idea that reducing equivalents are generated from cathodic electrons. Though we provide evidence for the generation of cellular energy carriers [i.e., ATP and NAD(P)H] in this system, there was no evidence of cell growth/division under these conditions. No growth was expected given that these experiments were performed in a system that lacked exogenous carbon inputs and several growth factors (i.e., vitamins and amino acids) that could provide carbon (55), and the *S. oneidensis* MR-1 genome contains no known complete carbon fixation pathway (38). However, the energy conserved in the form of ATP and cellular reducing power might plausibly support cell maintenance under cathodic conditions. As cathode biomass was maintained past the expected cell decay rates, it seems likely that the energy acquired is being invested in cellular upkeep. This is further supported by previous observations of *S. oneidensis* MR-1 that demonstrated that this organism requires energy in order to maintain cell surface attachment (56) and that such energy acquired under cathodic conditions might also be devoted to maintaining attachment. These observations support the proposal that MR-1 cells acquire energy for cellular processes under the studied cathodic conditions, energy that prolongs cell survival and/or allows maintenance of cell attachment.

The ability of *S. oneidensis* MR-1 to conserve energy under aerobic-cathode-oxidizing conditions may have important implications for the utility of this organism for microbe-electrode applications. The specifics of how electrons enter the cell and how this is coupled to a terminal electron acceptor affect cellular energy levels, the potential for growth, cell maintenance, and the overall process rates and long-term activities of these reactions. The environmental implications of these observations are more difficult to assess. In essence, are there environmental conditions under which *S. oneidensis* MR-1 can capitalize on the reversibility of the extracellular electron transport systems for acquiring energy for cell maintenance? *Shewanella* is an environmentally ubiquitous genus that is often found in redox transition zones or complex sediments (57). It is likely that this organism is commonly faced with shifts from anaerobic to aerobic conditions and/or carbon limitation. Though it appears to be a rate-adapted organism under carbon-replete anaerobic conditions (trading reaction rates for energetic efficiency), it may possess mechanisms for persistence in variable environments that are not presently well understood. Our results suggest the possibility that *S. oneidensis* MR-1, under carbon-limiting conditions in the presence of oxygen, capitalizes on solid-phase electron donors (e.g., sulfides and reduced iron species, possibly ones generated previously in the absence of oxygen) as electron sources for non-growth-linked energy acquisition. This may highlight an important ecologic advantage to such a reversible extracellular electron transport pathway, as has been suggested for iron cycling between iron-reducing and iron-oxidizing microbes (58). This work may also have implications for understanding electron uptake in organisms that can oxidize insoluble substrates (39, 58, 59), especially in the context of subsurface microbiology, as oxic, carbon-limited, and iron-rich (i.e., basalt) sediments are not uncommon in the deep sea (60, 61). Subsurface ecosystems often support orders of magnitude more microbes than should be allowable based on current energetic models and using the available organic carbon content (62). It has been postulated that lithotrophic interactions and especially lithoautotrophs might be part of this puzzle; however, when growth rates have been calculated, they are remarkably low (63). A non-growth-linked lithotrophic reaction might be a mechanism of potentially sustaining or slowing cell death and decay for subsurface microbes.

## MATERIALS AND METHODS

### Bacterial strains.

The bacterial strains used in this study are listed in Table 1. In the *SO-lux* strain constructed for this work, the *lux* operon was inserted into the *S. oneidensis* MR-1 genome via transposition at a neutral site via a mini-Tn*7*–*luxCDABE*–*tp* cassette (29), carrying both the *luxCDABE* operon behind the broad-host-range constitutive gammaproteobacterial promoter P1 (derived from a dihydrofolate reductase gene [64]) and the gene for trimethoprim resistance. A transposon-containing plasmid was transferred to *S. oneidensis* MR-1 via conjugation from *E. coli* (WMB026 cells), along with the PTNS3 plasmid, encoding a transposase, in the WMB026 strain. Viable *lux* mutant strains were isolated on LB media with trimethoprim (200 mg/liter) and screened for bioluminescence.

The in-frame gene deletion mutants utilized in this work were constructed and validated as described previously (65). In brief, primers were designed (listed in Table S1 in the supplemental material) to amplify the flanking regions of the gene targeted for deletion. These amplicons were then ligated into a suicide vector. After vector transfer into *S. oneidensis* MR-1, strains were screened for double recombinants as described previously (66).

10.1128/mBio.02203-17.10 TABLE S1  Primers used for gene deletion mutant construction as described in text. Download TABLE S1, DOCX file, 0.01 MB. Copyright © 2018 Rowe et al. 2018 Rowe et al. This content is distributed under the terms of the Creative Commons Attribution 4.0 International license.

### Cathodic culturing conditions.

Prior to electrochemical experiments, *S. oneidensis* MR-1 was grown in a batch at 30°C in standard media previously described, including Luria broth (LB) and a defined medium containing 10 mM lactate as the predominant carbon source (DM-lactate) (17). Conditioning of electrodes for chronoamperometry experiments was performed as described previously (17). In brief, DM-lactate-grown *S. oneidensis* MR-1 cells were added to electrochemical cells at an OD at 600 nm of 0.25 in fresh medium with 10 mM lactate. To induce cell attachment to electrodes, the working electrodes were poised at 397 mV versus the voltage of the SHE, while the reactors were purged with nitrogen to maintain anaerobic conditions. After 20 to 30 h of incubation, planktonic cells were removed from the reactors along with remaining medium. The attached cells were rinsed three times in carbon-free DM (CF-DM). Carbon-free DM lacked not only lactate but also the yeast extract added to traditional DM. The culture medium used in electrochemical experiments also lacked both trace metal and vitamin amendments for cathode experiments, as these can interact abiotically with electrodes. Unless otherwise indicated, cathodic conditions were applied to rinsed attached electrode biofilms in CF-DM by poising a working electrode potential of −303 mV versus that of the SHE and while reactors were being bubbled with room air at a continuous and constant rate (5 to 10 ml/min).

The following electron transport chain inhibitors were added to cathode experiments at the indicated concentrations: 5 mM potassium cyanide, 20 µM antimycin A, and 5 nM piericidin A. Various antibiotics, ampicillin (100 µg/ml), kanamycin (100 µg/ml), and trimethoprim (100 µg/ml), were used for cloning and selection marker purposes. The protonophore uncoupler CCCP was added at concentrations of 20 to 40 µM. During Lux cathode experiments, the only alteration to the aforementioned protocol was the addition of decanal to a final concentration of 0.002% to the electrochemical cells at the time points indicated by arrows in the figures. To ensure that no deleterious effects of solvents on *S. oneidensis* MR-1 cells occurred, we determined the growth curves of the *SO-lux* strain of *S. oneidensis* MR-1 amended with the highest concentration of DMSO used (0.05%, for the addition of RSG), as well as 0.002% decanal, and compared them to normal aerobic growth in DM-lactate (Fig. S8).

10.1128/mBio.02203-17.8 FIG S8  Growth of the *SO-lux* mutant is not effected by DMSO or decanal addition. Shown are aerobic *SO-lux* mutant growth curves (*n =* 3) in defined media with 7 mM lactate at 30°C in the absence or presence of 0.05% DMSO or 0.002% decanal. Error bars equal standard deviations of biological replicate OD measurements. Download FIG S8, EPS file, 1.1 MB. Copyright © 2018 Rowe et al. 2018 Rowe et al. This content is distributed under the terms of the Creative Commons Attribution 4.0 International license.

### Electrochemical conditions.

The electrochemical cell used for these experiments was described previously (67) and was constructed in-house. In brief, each reactor comprised a working electrode constructed from ITO-coated glass (SPD Laboratory, Inc., Hamamatsu Japan), a counterelectrode of platinum wire, and a reference electrode of Ag-AgCl in a 3 M KCl solution (constructed in our lab). The main reactor type maintained a liquid volume of 10 to 12 ml to an electrode surface area of 3.75 cm^2^. Electrochemical cells were designed for use with purchased ITO-plated glass coverslips (SPI, Westchester, PA) that had a 3.61-cm^2^ surface area to an 8-ml volume.

Experiments were performed using controlled voltage conditions and by measuring current production/consumption (i.e., chronoamperometry) through a potentiostat. The majority of chronoamperometry experiments were performed using eDAQ, quad channel potentiostats, and the corresponding eChart software (eDAQ Inc., Colorado Springs, CO); however, Lux chronoamerometry experiments were run on a Gamry 600 potentiostat using Framework software (Gamry, Warminster, PA) or a VMP3 potentiostat (BioLogic Company, France) using the parameters listed in the corresponding figure legends. Cyclic voltammetry was also performed using the Gamry 600 or the VMP3 potentiostat using the parameters listed in the corresponding figure legends.

### Fluorescence microscopy.

Electrode biofilms were visualized using an inverted microscope equipped with UV fluorescence detection. Cells were visualized using the lipid stain FM 4-64FX (Molecular Probes, Life Technologies, Inc.) at concentrations specified by the manufacturer. RedoxSensor Green (RSG) (Molecular Probes, Life Technologies, Inc.) studies were performed by adding approximately a 10 μM concentration of RSG in addition to lipid stain. Changes in fluorescence activity were monitored over time via microscopy. In brief, biofilm images were taken every 5 min over a period of 6 to 10 h, depending on the experiment. Fluorescence intensities between control (open-circuit), cathodic (applied reduced voltage), and inhibitor addition conditions were compared using approximately 80 images from each experimental state (including six different time points taken over a 30-min period). Cellular fluorescence intensities were compared for cells in each image using custom scripts in MatLab (codes are available upon request).

### ATP, ADP, and protein quantification.

Electrode biofilms were boiled for 10 min in nanopure water to release cellular ATP and ADP directly from electrode-attached cells. For protein recovery from electrode biofilms, samples were treated similarly, with the exception that 10 mM NaOH was added and samples were heated for at least 1 h at >85°C. Quantification of ATP and ADP was performed using the ATP/ADP ratio assay kit (Sigma-Aldrich Co.) according to the manufacturer’s specification (standard curves for ATP and ADP are provided in Fig. S9). Protein quantification was performed using the NanoOrange protein quantification kit (Molecular Probes, Life Technologies, Inc.) per the manufacturer’s specifications. Both luminescence and fluorescence were quantified on a BioTek (Winooski, VT) Synergy H4 microplate reader available through the USC Nanobiophysics core facility (http://dornsife.usc.edu/nanobiophysicscore/).

10.1128/mBio.02203-17.9 FIG S9  An ATP and ADP quantification assay supports accurate quantification of ATP and ADP. Standard curves (*n =* 3) were used for quantification of ATP (A) and ADP (B). Concentrations ranged from 5 nM to 50 µM for ATP and 1 nM to 10 µM for ADP, with detection limits approximately 1 order of magnitude lower than the lowest standard. Error bars indicate standard deviations of three individual standard curves. Download FIG S9, EPS file, 1.3 MB. Copyright © 2018 Rowe et al. 2018 Rowe et al. This content is distributed under the terms of the Creative Commons Attribution 4.0 International license.

### Quantification of *in vivo* luciferase activity.

Light emissions from an electrode biofilm were detected across an ITO-coated glass electrode using a photon multiplier tube and associated software (Photon Systems Inc., CA). Uncoupler experiments were performed using either the above-named systems or a Gene Light 55 GL-100A luminometer (Microtec, Japan). All experiments were conducted in a dark box to minimize light contamination.

Light emissions for cells grown under various lactate concentrations were quantified in the same reactor setups; however, the ITO-coated glass was replaced with a glass microscope slide (Fig. S2). For these experiments, an LB-grown *SO-lux* strain was diluted into a DM amended with 7 mM lactate. After ~12 h of growth, cells were rinsed and resuspended at an OD at 600 nm of 0.2 in media with various lactate concentrations (7, 0.7, and >0.07 mM lactate). Light production was measured after density normalization and averaged over a 20-min period.

### Statistical methods.

All experiments were performed in biological replicates (three experiments were carried out unless specified otherwise). Statistical analyses were performed using R and/or Excel. In experiments where quantitative comparisons were made, data were normalized to cell biomass using either protein content (in the case of current comparisons for mutants) or the total nucleotide pool (in the case of ATP values). In most cases, the cell populations for each experiment were constrained by the surface area of the electrode (380 mm^2^) and by ensuring surface attachment and planktonic cell removal through repeated washings. Manual cell counts were performed in ImageJ for time zero and time final biofilms for each experiment to ensure that population sizes remained consistent (a 20 to 25% standard deviation from the mean value measured for single populations). Twenty images from each biofilm (accounting for 0.1 mm^2^) demonstrated a normal sample distribution around the mean. Results of representative experiments and mean values and standard deviations of biological replicates are reported in the figure legends or in the text.
