# Supplementary material for: Tracking Electron Uptake from a Cathode into Shewanella Cells: Implications for Energy Acquisition from Solid-Substrate Electron Donors
Source: mBio. 2018 Feb 27;9(1):e02203-17. doi: 10.1128/mBio.02203-17 (PMC5829830; doi:10.1128/mBio.02203-17)
Supplement: TABLE S1 [file mbo001183695st1.docx]

*Supplementary Materials for*:

Tracking electron flow from a cathode to a *Shewanella* cell: implications for generating maintenance energy from solid substrates.

Annette Rowe, Pournami Rajeev, Abhiney Jain, Sahand Pirbadian, Akihiro Okamoto, Jeffrey A. Gralnick, Moh El-Naggar, Kenneth Nealson

**Table S1.** Primers used for gene deletion mutant construction as described in text.

| **Name** | **Gene deletions** | **Upstream Primers** | **Downstream Primers** |
| --- | --- | --- | --- |
| *dms-*all | SO1427-1432 | F-5’-GTACGGATCCGGATACCTTACTGCGCCTAATG-3’ | F-5’-GACTACTAGTGGTGGATAAATAAATTCGAGTG-3’ |
|  |  | R-5’-GTACACTAGTCCATCTCATATTTAATGCCTTATGG-3’ | R-5’-GACTGAGCTCGAAATGACGGAACAGTTGATTACCG-3’ |
| *petABC* | SO0608-0610 | F-5’-ACGAACTAGTCTCCTATGTGAAGTGGCGT-3’ | F-5’-TTTTGGGCCCAGGCACCAATAACGGGTA-3’ |
|  |  | R-5’-ATTTGGGCCCCGCATTGCTCATCCACTT-3’ | R-5’-TAGAGAGCTC GCCACAATCGAAGCCAT-3’ |
| *cox-*all | SO2361-2346 | F-5’-GCATGGATCCAATTGCAGATGTGTTC-3’ | F-5’-GCATACTAGTAGCTTGTCAAACAAGT-3’ |
|  |  | R-5’-GCATACTAGTCTGGGAATGGTTCATC-3’ | R-5’-GCATGAGCTCGGGTGGTGGAGTTGA-3’ |
|  | SO3285-3286 | F-5’-GCATGGATCCGCCCCCAACTCAATGA-3’ | F-5’-GCATACTAGTCTCTATTAGAACTAAG-3’ |
|  |  | R-5’-GCATACTAGTAATCATCGGTGACTCC-3’ | R-5’-GCATGAGCTCCATCAAGCCGTTGATA-3’ |
|  | SO4606-4609 | F-5’-GCATGGATCCTCACCCATTAAAGTCG-3’ | F-5’-GCATACTAGTGTACTTTAAAACAATAT-3’ |
|  |  | R-5’-GCATACTAGTCTTCACAAGACTTCTC-3’ | R-5’-GCATGAGCTCGTGCCGACAAGGGGA-3’ |
| *nuo* | SO1018, partial SO1017&SO1019 | F-5’-ACTAGTCGGTAACGAGAATGAGGA-3’ | F-5’-GGCGCGCCAAGACAGGCTACTGAT-3’ |
|  |  | R-5’-GGCGCGCCCTTCGATCATTTGAA-3’ | R-5’-TGATAACCGCAAGTCCATGAGCTC-3’ |
